# Revisiting the Role of *ß*-Tubulin in *Drosophila* Development: *β-tubulin60D* is not an Essential Gene, and its Novel *Pin* ^*1*^ Allele has a Tissue-Specific Dominant-Negative Impact

**DOI:** 10.3389/fcell.2021.787976

**Published:** 2022-01-17

**Authors:** Ramesh Kumar Krishnan, Naomi Halachmi, Raju Baskar, Anna Bakhrat, Raz Zarivach, Adi Salzberg, Uri Abdu

**Affiliations:** ^1^ Department of Life Sciences, Ben-Gurion University of the Negev, Beer’Sheva, Israel; ^2^ Department of Genetics and Developmental Biology, Rappaport Faculty of Medicine, Technion, Haifa, Israel; ^3^ National Institute for Biotechnology in the Negev and the Ilse Katz Institute for Nanoscale Science and Technology, Ben-Gurion University of the Negev, Beer Sheva, Israel

**Keywords:** bristle, drosophila, tissue-specific, tubulin, tubulin isotypes

## Abstract

Diversity in cytoskeleton organization and function may be achieved through alternative tubulin isotypes and by a variety of post-translational modifications. The *Drosophila* genome contains five different *β-tubulin* paralogs, which may play an isotype tissue-specific function *in vivo*. One of these genes*,* the *β-tubulin60D* gene, which is expressed in a tissue-specific manner, was found to be essential for fly viability and fertility. To further understand the role of the *β-tubulin60D* gene, we generated new *β-tubulin60D* null alleles (*β-tubulin60D* ^*M*^) using the CRISPR/Cas9 system and found that the homozygous flies were viable and fertile. Moreover, using a combination of genetic complementation tests, rescue experiments, and cell biology analyses, we identified *Pin* ^*1*^, an unknown dominant mutant with bristle developmental defects, as a dominant-negative allele of *β-tubulin60D*. We also found a missense mutation in the Pin^1^ mutant that results in an amino acid replacement from the highly conserved glutamate at position 75 to lysine (E75K). Analyzing the *ß*-tubulin structure suggests that this E75K alteration destabilizes the alpha-helix structure and may also alter the GTP-Mg^2+^ complex binding capabilities. Our results revisited the credence that *β-tubulin60D* is required for fly viability and revealed for the first time in *Drosophila*, a novel dominant-negative function of missense *β-tubulin60D* mutation in bristle morphogenesis.

## 1 Introduction

Microtubules are polymers of *α*/*β* tubulin subunits, and they carry out a wide range of functions in eukaryotes (Desai and Mitchison 1997; Nogales 2001; Goodson and Jonasson 2018). The expression of different tubulin isotypes can vary according to cell type and stage of development (Ludueña 2013). The *Drosophila β-tubulin* gene family includes five members, each expressed in a unique pattern based on developmental timing and tissue-type specificity (Fyrberg and Goldstein 1990). The most divergent *ß*-tubulin paralogs (*β-tubulin* 85D and *ß-tubulin* 65B) are expressed exclusively in testis. *ß-tubulin* 85D, but not *ß-tubulin* 65B, has been characterized in considerable detail; it is required in the germline for male meiotic divisions and sperm axoneme formation (Kemphues et al., 1982; Fuller et al., 1988). *β-tubulin56D* is maternally supplied to the embryo and zygotically expressed during neurogenesis and in muscle attachment sites shortly after the insertion of muscles into the epidermis (Kemphues et al., 1982; Bialojan et al., 1984; Buttgereit et al., 1991; Buttgereit et al., 1996). It was shown that *β-tubulin 56D* is required for myoblast fusion, myotube elongation, and sarcomere formation during *Drosophila* embryogenesis (Rudolf et al., 2012). Recently, it was shown that the function of *ß-tubulin* 97EF is dispensable for viability and fertility, but it has a tissue-specific requirement for regulation of MT stability in a temperature-dependent manner (Myachina et al., 2017). The expression of *β-tubulin60D* is also tissue-specific; during embryogenesis, *β-tubulin60D* expression starts in differentiating mesodermal cell types and occurs in chordotonal organs, imaginal discs, and somatic cells of the adult gonads Halachmi et al. is (Dettman et al., 2001). An extensive study on the role of *β-tubulin60D* led to the identification of multiple alleles of *β-tubulin60D*, which showed lethality at different stages of development, from embryogenesis to larval stages (Kimble et al., 1990; Dettman et al., 1996; Dettman et al., 2001).

Microtubules serve both as a scaffold for intracellular transport and contribute to cell polarity (Meiring et al., 2020). The elongated *Drosophila* bristle is a single polyploid, highly polarized cell with a distinct direction of growth and a cone-like shape (Lees and Waddington 1942; Tilney et al., 2004). The polarized *Drosophila* mechanosensory bristle cytoplasm is filled with short MTs that constitute a significant component of the shaft cytoplasm. These MTs appear to be stable during development and shorter in length than the mature bristle shaft (Tilney et al., 2000). MT organization in bristles revealed two populations of MTs: one population is stable and uni-polarized, organized with their minus-end toward the bristle tip (Bitan et al., 2010), and believed to serve as polarized tracks for proper organelle and protein distribution (Tilney et al., 2000). The second MT population is dynamic with mixed polarity and contributes to proper axial growth (Fei et al., 2002; Bitan et al., 2010), probably establishing bristle polarity (Bitan et al., 2012).

This study reveals that *β-tubulin60D* is not an essential gene, as was described before, and elucidates a tissue-specific role of one of the *β-tubulin* paralogs, *β-tubulin60D,* in bristle MT assembly. We identified *Pin* ^*1*^ as a dominant-negative allele of *β-tubulin60D,* which explicitly affects bristle development. Using sequencing and structural analysis, we demonstrated that the *Pin* ^*1*^ mutation is caused by a single amino acid substitution, which affects the GTP-Mg^2+^ complex binding and interferes with the alpha helix’s stability.

## 2 Materials and Methods

### 2.1 *Drosophila* Stocks

Oregon-R was used as the wild-type control. The following mutant and transgenic flies were used: *Suppressor of Hairless*, *Su(H)* (Nagel et al., 2017), Df(2R)Exel6082 (Bloomington *Drosophila* Stock Center #7561), *nervy* ^*PDFKG38*^ and *nervy* ^*PDFKG1*^ (Terman and Kolodkin 2004), and *Pin* ^*1*^ (Bloomington *Drosophila* Stock Center). For the rescue experiment, *M{UAS-βTub60D.ORF}ZH-86Fb* was used (Myachina et al., 2017). Bristle-specific expression was induced under the control of the *neur-*Gal4 driver (for the rescue experiment) or *sca-*Gal4 driver. All of the Gal4 lines were obtained from the Bloomington *Drosophila* Stock Center.

### 2.2 Developmental Staging and Pupal Dissection

Stages of all flies were determined from puparium formation (Bainbridge and Bownes, 1981). White prepupae were collected and placed on double-sided scotch tape in a petri dish placed in a 25°C incubator, as previously described (Tilney et al., 2000). At the appropriate time of incubation (36–44 h APF, unless indicated otherwise), the pupae were dissected for live imaging, fixation, and proteomic screening. The pupal case was removed as described in (Fukutomi et al., 2018). After removing the pupal case, the pupae were dissected as described elsewhere in detail (Tilney et al., 2000).

### 2.3 Bristle Phalloidin and Antibody Staining

Bristle fixation and staining were performed as previously described (Guild et al., 2003; Melkov et al., 2015). Confocal images were taken using an Olympus FV1000 laser scanning confocal microscope and are shown here as z-projections in a few optical frames that covered the bristle cell. Primary antibodies used were anti-α-acetylated tubulin mouse monoclonal antibodies (1:250; Sigma, T7451) and anti-β-tubulin mouse monoclonal antibodies (1:250; Sigma T5201). Bristles from CRISPR KO flies were stained with anti-β3Tub (called in this paper- *β-tubulin60D*) polyclonal rabbit (1:1,000) (Leiss et al., 1988). Cy3-conjugated goat anti-mouse (1:100; Jackson ImmunoResearch) secondary antibody was used. For actin staining, Oregon Green 488- or Alexa Fluor 568-conjugated phalloidin (1:250; Molecular Probes) was used.

### 2.4 Scanning Electron Microscopy

Adult *Drosophila* flies were fixed and dehydrated by immersing them in increasing concentrations of ethanol (25, 50, 75%, and twice in 100%; 10 min each). The flies were then completely dehydrated using increasing concentrations of hexamethyldisilazane (HMDS) in ethanol (50%, 75%, and twice in 100%; 2 h each). The samples were air-dried overnight, placed on stubs, and coated with gold. The specimens were examined with a scanning electron microscope (SEM; JEOL model JSM-5610LV). Length measurements of adult bristles were performed using Image J (version 1.52t) software with the straight-line tool. This tool allows the creation of line selections and then the calculation of the length of these lines. To test for differences in bristle length and width between the wild-type and the different mutants, we used a one-way analysis of variance (ANOVA) followed by a Tukey analysis.

### 2.5 Sample Preparation for Mass Spectrometry Analysis

The pupal case was removed as described in (Fukutomi et al., 2018). Then the pupae were dissected as described elsewhere in detail (Tilney et al., 2000). The dissection procedure resulted in the isolation of thorax dorsal side tissue, which was then cleaned of interior organs and fat particles as described in (Tilney et al., 2000). All procedures were conducted in phosphate-buffered saline (PBS). The head and abdomen parts of the tissue were cut, leaving only the thorax intact, which was then put in a vial of PBS with a protease inhibitor cocktail (Sigma). Each group consisted of triplicates of 20 thoracic tissues.

### 2.6 Proteolysis and Mass Spectrometry Analysis

The samples were ground in 10 mM DTT 100 mM Tris and 5% SDS, sonicated, and boiled at 95°C for 5 min. They were then precipitated in 80% acetone. The protein pellets were dissolved in 9 M Urea and 100 mM ammonium bicarbonate and reduced with 3 mM DTT (60°C for 30 min), modified with 10 mM iodoacetamide in 100 mM ammonium bicarbonate (room temperature for 30 min in the dark), and digested in 2 M Urea25 mM ammonium bicarbonate with modified trypsin (Promega), overnight at 37°C in a 1:50 (M/M) enzyme-to-substrate ratio. The resulting tryptic peptides were desalted using C18 tips (Harvard), dried, and re-suspended in 0.1% formic acid. They were analyzed by LC-MS/MS using a Q Exactive Plus mass spectrometer (Thermo) fitted with a capillary HPLC (easy nLC 1000, Thermo). The peptides were loaded onto a homemade capillary column (20 cm, 75 micron ID) packed with Reprosil C18-Aqua (Dr. Maisch GmbH, Germany) in solvent A (0.1% formic acid in water). The peptide mixture was resolved with a (5–28%) linear gradient of solvent B (95% acetonitrile with 0.1% formic acid) for 180 min, followed by a 15-min gradient of 28–95% and 15 min at 95% acetonitrile with 0.1% formic acid in water at flow rates of 0.15 μl/min. Mass spectrometry was performed in a positive mode using repetitively full MS scanning followed by high collision-induced dissociation (HCD, at 25 normalized collision energy) of the ten most dominant ions (>1 charges) selected from the first MS scan. The mass spectrometry data were analyzed using the MaxQuant software 1.5.2.8. (www.maxquant.org). using the Andromeda search engine, searching against the *Drosophila* UniProt database with a mass tolerance of 6 ppm for the precursor masses and 6 ppm for the fragment ions. Peptide- and protein-level false discovery rates (FDRs) were filtered to 1% using the target-decoy strategy. Protein tables were filtered to eliminate the identifications from the reverse database and common contaminants and single peptide identifications. The data were quantified by label-free analysis using the same software, based on extracted ion currents (XICs) of peptides, enabling quantitation from each LC/MS run for each peptide identified in the experiments. Statistical analysis of the identification and quantization results was done using Perseus 1.6.7.0 software.

### 2.7 Generation of *β-tubulin60D* Knockout Flies by CRISPR Cas-9-Mediated Genome Editing

To generate knockout flies, two guide RNA sequences were identified (sgRNA1 - GGC​GGT​CCC​GTC​TCC​AAA​GGG​GG & sgRNA2 - GGA​GCC​CGG​AAC​CAT​GGA​GTC​GG) at http://targetfinder.flycrispr.neuro.brown.edu/and cloned into plasmid pU6-BbsI-chiRNA. Then 1 Kb sequence stretches upstream and downstream of *β-tubulin60D* were cloned into the donor pHD-DsRed-attP vector. Finally, injection of both vectors and fly screening was carried out by BestGene. To molecularly verify that our Knock in construct replaced the exon, WT and CRISPR mutant *Drosophila* genomic DNA (gDNA) was extracted using PureLink™ Genomic DNA Mini Kit following manufacturer’s instructions (Invitrogen). PCR amplifications were performed using the following forward primer: 5′-GTG​CTG​AAG​GGC​GAG​ATC​C-3′ and reverse primer 5′- CCA​CCA​GCT​CGG​CGC​CCT​C-3′. The PCR amplification was as follows: 95 °C for 3 min; 35 cycles of 95°C for 15 s, 65°C for 15 s, and 72°C for 30 s, with a final extension step of 72°C for 2 min. The PCR products were analyzed by gel electrophoresis and sequenced. .

### 2.8 Fertility Assay

Three virgin *β-tubulin60D* ^*M*^ females were mated with two wild-type (WT) males, and two *β-tubulin60D* ^*M*^ males were mated with three virgin WT females in a vial containing yeast for 2 days. Matings were performed in triplicate for each genotype. The flies were transferred to new vials containing fresh yeast and were let to lay eggs for 1 day. The flies were then discarded, and the adult progeny were collected and counted after 10 days at 25°C. The progeny per female and the average number and standard deviation of progeny per genotype were calculated from each vial. Finally, a percentage of relative fertility was calculated (Spracklen et al., 2014).

### 2.9 Chordotonal Organ Staining/Cuticle Preparation

Seven larvae from each genotype were dissected and immune-stained as previously described (Halachmi et al., 2012). Mouse anti-αtubulin-85E (1:20) (Nachman et al., 2015) was used for visualizing the LCh5 accessory cells and rabbit anti-*βTub60D* (1:1,000) (Leiss et al., 1988) for verifying the loss of *βTub60D* in the mutant larvae. Secondary antibodies were Cy3-conjugated anti-rabbit and Cy5-conjugated anti-mouse (1:100, Jackson Laboratories, Bar-Harbor, Maine, United States). Stained larvae were mounted in DAKO mounting medium (DAKO Cytomation, Denmark) and viewed using confocal microscopy (Axioskop and LSM 510, Zeiss). To guarantee similar age of all tested larvae, flies of all genotypes were let to lay eggs for 3 h, and the progeny were aged for 118–121 h at 24°C. For each chordotonal organ, we measured the length of the cap plus cap-attachment cells, the ligament plus ligament-attachment cells, and the space between the cap and ligament cells, which represents the scolopale cells. The length of each cell was normalized to the total length of the organ.

### 2.10 Data Analyses

Quantitative data are expressed as mean ± standard error of the mean (SEM). All the statistical analyses were performed using a one-way ANOVA, and *p-*values ≤ 0.05 were considered significant for all analyses. Statistical significance was checked with a pairwise post-hoc Tukey HSD. All the statistical analyses were performed using STATISTICA, version 10.

## 3 Results

### 3.1 Proteomic Analysis Led to Identification of *β-tubulin60D* as Bristle-Specific Gene

**FIGURE 1:**
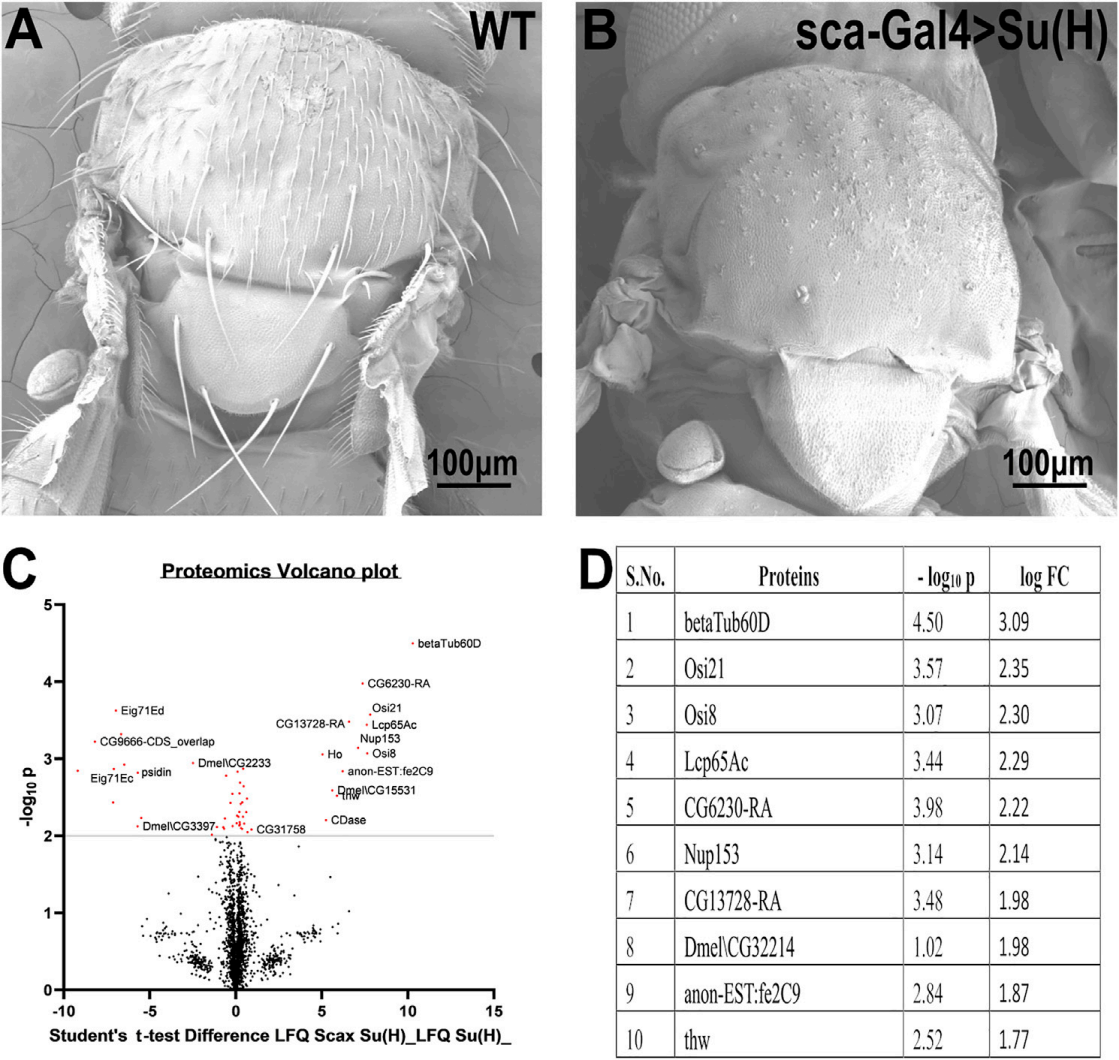
Proteomic profiling in the thoracic tissue of *Drosophila*. Scanning electron micrographs of the thorax wild-type **(A)** and *sca*-Gal4>Su (H). **(B)** Suppressor of Hairless [Su (H)], a transcriptional regulator in the notch signaling pathway, when driven by a *sca*-Gal4 driver, results in the complete absence of both microchaeta and macrochaeta. **(C)** Volcano plot showing differentially expressed proteins between WT (control) and Su (H); *sca*-Gal 4 (test) groups. Proteins with statistically significant differential expression (−log_10_ *p* > 2.0) are located in the top right and left quadrants. **(D)** Quantitative proteomics table of proteins with differential abundance in biological triplicates. Twenty-seven proteins were found to be downregulated as compared to the wild-type. The table shows the first ten proteins with their−log10 *p*-values and corresponding log-fold change values. *ß*-tubulin60D was chosen as a candidate protein because of its highly significant *p*-value.

To identify genes that may be involved in bristle development, we compared the repertoire of proteins of Su(H); *sca*-Gal4 flies, which lack all their bristles, versus wild-type flies (Figure 1A). This comparative proteomic analysis/approach allowed us to identify bristle-specific proteins. We generated flies lacking bristles on their thorax by upregulating Su(H) expression specifically in the bristle lineage. Su(H) overexpression resulted in a complete loss of bristle cells (both microchaeta and macrochaeta) and the formation of extra socket cells (Schweisguth and Posakony 1994) (Figure 1B). Proteins were extracted from thoraces of wild-type flies and Su (H) overexpressing, digested by trypsin, and analyzed by LC-MS/MS on Q Exactive Plus (Thermo). Samples were prepared/analyzed in triplicates for statistical significance. The complete list of differentially expressed proteins is presented in Supplementary File 1. Specifically, our proteomic analysis identified 27 proteins that were significantly downregulated in the flies expressing Su(H) as compared to wild type (Figures 1C,D). Among these differentially expressed proteins, *ß*-tubulin60D showed the most significant (*p*-value < 3.18E^−05^) fold change. We, therefore, decided to study the role of *ß*-tubulin-60D in bristle development.

### 3.2 Loss of *β-tubulin60D* Does not Lead to Lethality or Sterility and Does not Impair Bristle Development and ChO Morphogenesis

**FIGURE 2:**
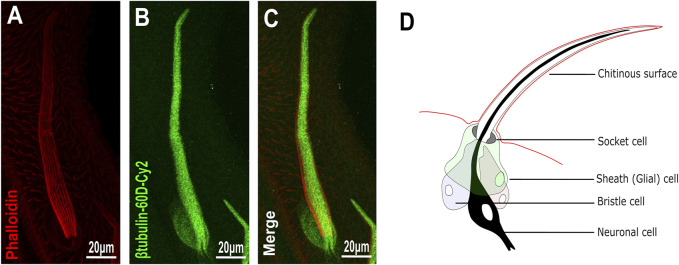
Expression of *β-tubulin60D* in bristle. Confocal projections of bristles of ∼38 h APF from wild-type **(A–C)** pupae stained with Oregon red-phalloidin (red) and with anti-*β*-*tubulin-60D* antibodies (green). In wild-type bristles, *ß*-*tubulin60D* is abundant along the entire bristle shaft. APF–after prepupa formation. **(D)** Schematic diagram of the mechanosensory bristle. Each bristle sensory organ is composed of 4 cells–socket cell (tormogen), bristle cell (trichogen), neuronal cell and sheath cell.

To analyze the distribution/expression pattern of *ß*-tubulin60D protein within the bristle lineage, we immunostained pupal thoraces using *ß*-tubulin60D-specific antibodies. This staining revealed that *ß*-tubulin60D protein is distributed along the entire bristle shaft but is excluded from the other lineage-related cells, namely, the socket, neuron, and sheath cells (Figures 2A–C).

**FIGURE 3:**
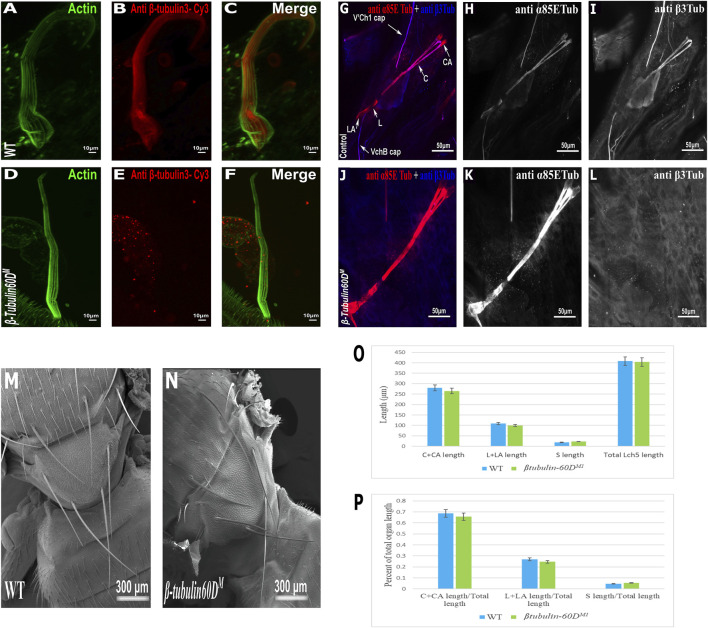
*β-tubulin60D* null allele shows the absence of β-tubulin60D protein in both bristles and chordotonal organs. Confocal projections of bristles of ∼38 h APF from wild-type **(A–C)** and *β-tubulin60D* ^*M*^ **(D–F)** pupae stained with Oregon green-phalloidin (green) and with anti *ß*-*tubulin60D* (also called *β*3-tubulin antibodies) (red). In wild-type bristles, *ß*-*tubulin-60D* is abundant along the entire bristle shaft, whereas in *β-tubulin60D* ^*M*^ flies, there is a complete absence of the *ß*-*tubulin60D* protein. APF–after prepupa formation. Immunostaining of an LCh5 organ of a third instar WT **(G–I)** and *ß*-*tubulin-60D* CRISPR KO **(J–L)** larvae by double staining with anti-*α*85ETub antibodies (in red) and anti-*β*-*tubulin60D* (in blue). Anti-*β*-*tubulin60D* is expressed explicitly in cap cells **(I)**, whereas it is not present in the cap cells of *β-tubulin-60D* CRISPR KO lines **(L)**. *β-tubulin60D* ^*M*^ mutants show normal bristle development. Scanning electron micrograph of adult bristles from wild-type flies **(M)** and *β-tubulin60D* ^*M*^ **(N)**. The CRISPR mutants do not show any visible structural defects in the bristle and appear similar to WT bristles. *β-tubulin60D* does not play a significant role in chordotonal organ morphogenesis. The length of the different ChO cells of LCh5 organs of *β-tubulin60D* ^*M1*^ homozygous third instar larvae was measured and compared to the wild-type larvae. **(O)** The graph shows the average length (in µm) of the cap (C) + cap-attachment cells (CA), ligament (L) + ligament-attachment cells (LA), and space (S) between the cap cells and ligament cells (this space corresponds to the scolopale cell). **(P)** The length of each cell was normalized to the total length of the organ. No significant difference is seen in both homozygous and heterozygous *β-Tubulin60D* compared to the wild-type larvae.

To test whether *β-tubulin60D* is involved in bristle development, we generated a *β-tubulin60D* null allele using CRISPR/Cas9-mediated genome editing. For our CRISPR experiment, we designed two sgRNAs to replace the second exon by inserting the visible marker 3 X-P3-dsRed (Supplementary Figure S1A). Five independent mutated knock-in insertion *Drosophila* lines were generated and named: *β-tubulin60D* ^*M1*^ to *β-tubulin60D* ^*M5*^. First, we confirmed that all the five mutated lines contained the 3 X-P3-dsRed, which replaced the entire second exon (Supplementary Figure S1B). To verify that indeed *β-tubulin60D* ^*M*^ is a protein null allele, we stained homozygous larvae and pupae and examined *β-tubulin60D* expression in the chordotonal organ and bristle. As described above, usually, *ß*-tubulin60D protein is expressed both in the bristle shaft (Figures 3A–C) and in the cap cell of the ChO (Figures 3G–I). However, in the *β-tubulin60D* ^*M*^ mutant, no expression was detected in the bristle (Figures 3D–F) or in the ChO cap cells (Figures 3J–L), confirming that we had generated a complete loss of function allele of the *β-tubulin60D* gene. Previously, it was published that the *β-tubulin60D* gene is essential for viability and fertility (Kimble et al., 1990). In contrast to this published data, we found that all our *β-tubulin60D* ^*M*^ alleles were viable, and both males and females were fully fertile (Supplementary Table S1).

The specific expression of *β-tubulin60D* in the bristle shaft cells and its dramatic down-regulation protein in bristle-less flies could point to a possible role of this gene in bristle development. To address this issue, we examined bristle morphology in *β-tubulin60D* ^*M*^ flies by scanning electron microscopy. Surprisingly, no apparent defects were detected in the bristles of the *β-tubulin60D* ^*M*^ mutant/homozygous flies (Figures 3M,N).

In the ChO, β-tubulin60D is expressed solely in the cap cell—the only cell type within the ChO lineage that elongates dramatically during larval growth. Moreover, based on the similarity between the lineages of ChOs and the external sensory organs/bristles, the cap cell parallels the shaft cell (Lai and Orgogozo 2004). The cap cell transmits muscle-generates forces from the cuticle to the sensory neuron (Hassan et al., 2019). We investigated the possible role of *β-tubulin60D* in cap cell elongation by measuring the length of the different ChO cells in wild-type versus mutant third instar larvae. We found that, on average, the length of cap plus cap-attachment cells in wild-type larvae was 280 ± 38.9 μm (*n* = 42), constituting 68.5% of the organ’s total length. In *β-tubulin60D* ^*M1*^ homozygous larvae, no significant change in the length of the cap plus cap-attachment cells was noticed; 265.5 ± 39.9 μm (*n* = 59), constituting 68.9% of the organ’s total length (Figures 3O–P). These results suggest that *β-tubulin60D* does not play a significant role in cap cell elongation and ChO morphogenesis.

### 3.3 *Pin* ^*1*^ Is *a* Dominant-Negative Allele of *β-tubulin60D*, Which Affects Bristle but not ChO Development

**FIGURE 4:**
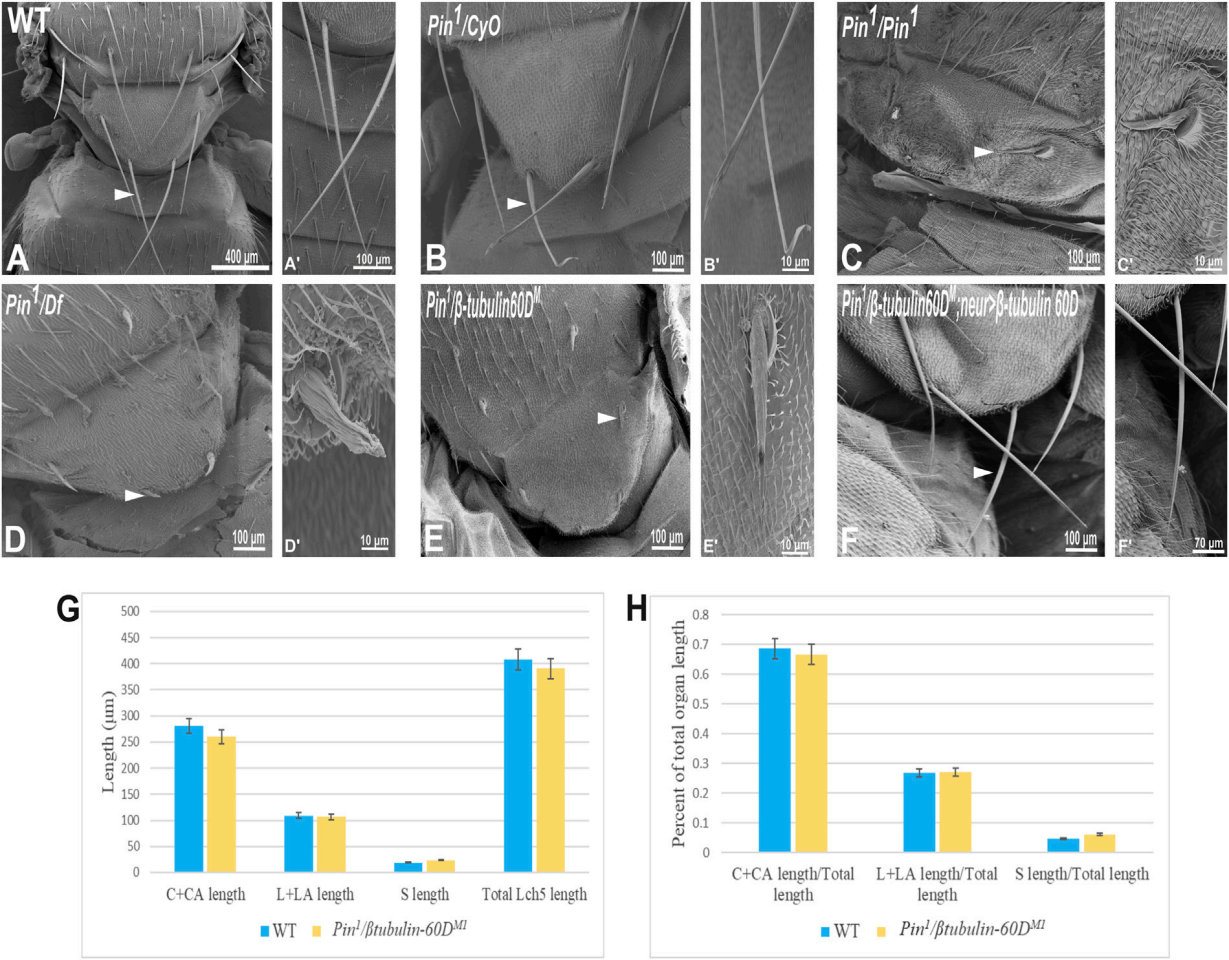
Gene mapping of *Pin* ^*1*^ bristle phenotype. Scanning electron micrograph of adult bristles from wild-type flies **(A)**, *Pin* ^*1*^/CyO **(B)**, *Pin* ^*1*^/*Pin* ^*1*^ **(C)**, *Pin* ^*1*^/Df **(D)**, *Pin* ^*1*^/*β-tubulin60D* ^*M*^ **(E)**, and *Pin* ^*1*^/*β-tubulin60D* ^*M*^; *neur*>*β-tubulin60D*. The *Pin* ^*1*^ bristle **(B′)** is shorter compared to wild-type bristles **(A′)**. Also, *Pin* ^*1*^/Df bristle **(D′)** and *Pin* ^*1*^/*β-tubulin60D* ^*M*^ **(E′)** bristles are comparatively smaller than *Pin* ^*1*^ and wild-type bristles. *Pin* ^*1*^ and *Pin* ^*1*^/Df have abnormally organized surface grooves. *Pin* ^*1*^/*β-tubulin60D* ^*M*^ bristles show smoother surfaces. *β-tubulin60D* rescues the bristle phenotype of *Pin* ^*1*^. **(F)** shows the rescued bristle phenotype by *β-tubulin60D*, resulting in longer bristles with properly tapered tips **(F′)** just like the wild-type bristles. Thus, the expression of *β-tubulin60D* using *neur*-Gal4 rescues the *Pin* ^*1*^ bristle phenotype. Arrowheads point to the bristle, which is shown as a higher magnification image. The length of the different ChO cells of LCh5 organs of *Pin* ^*1*^ */β-tubulin60D* ^*M1*^ transheterozygous third instar larvae was measured and compared to the wild-type larvae. **(G)** The graph shows the average length (in µm) of the cap (C) + cap-attachment cells (CA), ligament (L) + ligament-attachment cells (LA), and space (S) between the cap cells and ligament cells (this space corresponds to the scolopale cell). **(H)** The length of each cell was normalized to the total length of the organ. No significant difference is seen in both homozygous and heterozygous *β-tubulin60D* compared to the wild-type larvae.

In parallel to the generation of the *β-tubulin60D* null allele, we searched known but uncharacterized mutations that cause abnormal bristle phenotypes for mutations that map to the genomic region in the vicinity of the *β-tubulin60D* gene (thus representing candidate alleles of *β-tubulin60D*). One such bristle defective mutant is called *Pin*. In the heterozygous *Pin* ^*1*^ allele*,* macrochaeta, but not microchaeta, are shortened and sharply tapered at the tip (Figures 4B,B') compared to wild-type bristles (Figures 4A,A'). The length of macrochaeta from heterozygous *Pin* ^*1*^ mutant (Figure 4B) measured 224.87 ± 16.9 µm, which was significantly shorter (*p* < 0.01) as opposed to the wild-type, which had a bristle length of 395.79 ± 1.9 µm (Table 1). Homozygous *Pin* ^*1*^ mutants (Figures 4C,C') die as pharate adults, and the average length of their bristles is significantly shorter (40.73 ± 4.2 µm) than the heterozygous *Pin* ^*1*^ mutant.

**TABLE 1:** Table showing the length, base width, and tip width of wild-type along with the homozygous, heterozygous, hemizygous, and transheterozygous *Pin* ^*1*^ mutants.

S.No	Genotype	Length (µm)	Base width (µm)	Tip width (µm)
1	WT	395.79 ± 1.9^a^	9.90 ± 0.9^a^	3.87 ± 0.2^a^
2	*Pin* ^*1*^ */Pin* ^*1*^	40.73 ± 4.2^b^	6.99 ± 0.4^b^	1.90 ± 0.2^b^
3	*Pin* ^*1*^/CyO	224.87 ± 16.9^c^	11.20 ± 0.5^a^	4.29 ± 0.2^a^
4	*Pin* ^*1*^/Df	57.57 ± 5.6^d^	9.75 ± 0.5^a^	2.77 ± 0.1^a^
5	*Pin* ^*1*^; *β-tubulin60D* ^*M*^	76.57 ± 6.3^f^	8.53 ± 0.40^a^	2.11 ± 0.21^c^
6	*Pin* ^*1*^/*β-tubulin60D* ^*M*^; *neur*> *β-tubulin60D*	328.17 ± 8.6^a^	8.75 ± 0.1^a^	3.19 ± 0.2^a^

Tukey’s test for post-hoc analysis shows that bristle length is statistically significant to each other (*p* < 0.01) compared to WT. When the transheterozygous mutant is rescued with a wild-type *β-tubulin60D*, the bristle length equals almost that of the WT bristles. Tukey’s test for post-hoc analysis shows that bristle length is the same statistically, thereby showing that *β-tubulin60D* rescues the bristle phenotype in the mutants. Ten SEM micrographs of the thoracic tissue were taken for analysis, and a minimum of five bristles was taken for measurement.

^a-d^ Different letters in the same column show that they are statistically significant compared to the wild-type, whereas the same letter in the column shows no significance.

To map the *Pin* ^*1*^ allele, we first used deficiency mapping and found that Df(2R)Exel6082, which lacks the genomic region 60C4 to 60C7, fails to complement the *Pin* ^*1*^ allele, as demonstrated by the effect on bristle development where the length of the hemizygous allele was 57.57 ± 5.6 µm (Figures 4D,D'), similar in their length to the *Pin* ^*1*^ homozygous flies. Also, we found that the hemizygous flies, *Pin* ^*1*^/Df, are viable, which means that the lethality of *Pin* ^*1*^ homozygotes is probably due to other mutations in the background of the stock. Next, in order to find a smaller genomic region that will fail to complement the bristle phenotype of *Pin* ^1^, we used two *nervy* alleles, *nervy* ^*PDFKG1*^ and *nervy* ^*PDFKG38*^, that remove the genomic region between the following P-elements: *KG(2)06386* and *KG(2)04837* (Terman and Kolodkin 2004). Since these deficiencies fail to complement the *Pin* ^*1*^ allele bristle defects, it suggests that *Pin* could be an allele of one of 11 genes, among them *β-tubulin60D*. To test whether *Pin* ^*1*^ is a dominant allele of *β-tubulin60D*, we crossed *Pin* ^*1*^ with our *β-tubulin60D* ^*M1*^ allele and found that *trans*-heterozygous flies had shorter bristles (76.57 ± 6.3 µm) similar in their length to both hemi- and homozygous *Pin* ^*1*^ mutants (Figures 4E,E'). These results suggest that *Pin* ^*1*^ could be a dominant allele of the *β-tubulin60D* gene. To further characterize the nature of the *Pin* ^*1*^ allele and to verify whether it is a dominant-negative or a neomorph allele of the *β-tubulin60D* gene, a rescue experiment was conducted. We generated transgenic flies that over-express the *ß*-tubulin60D protein in the bristle using the *neur-*Gal4 driver in a *trans*-heterozygous mutant background (*Pin* ^*1*^/*β-tubulin60D* ^*M1*^). The rescue experiment demonstrated that over-expression of *β-tubulin60D* completely rescued the short-bristle phenotype detected in *Pin* ^*1*^/*β-tubulin60D* ^*M1*^ flies (Table 1) (Figures 4F,F'), suggesting that indeed *Pin* ^*1*^ is a dominant-negative allele of *β-tubulin60D*.

Since the phenotypical analysis of the *Pin* ^*1*^ allele implicates *β-tubulin60D* as required for bristle development, we tested whether this allele affects ChO morphogenesis. To test whether the *Pin* ^*1*^ mutation also affects ChO development, we characterized the ChOs of third instar larvae of *Pin* ^*1*^ */β-tubulin60D* ^*M1*^ larvae and compared them to WT larvae. This analysis showed that in *Pin* ^*1*^ */β-tubulin60D* ^*M1*^ larvae, the length of the cap plus cap-attachment cells was 260.4 ± 29.4 μm (*n* = 44), constituting 66.6% of the organ’s total length, which is not significantly different from WT larvae (Figures 4G,H). This observation suggests that the *Pin* ^*1*^ allele has a tissue-specific effect impairing only macrochaeta development.

**FIGURE 5:**
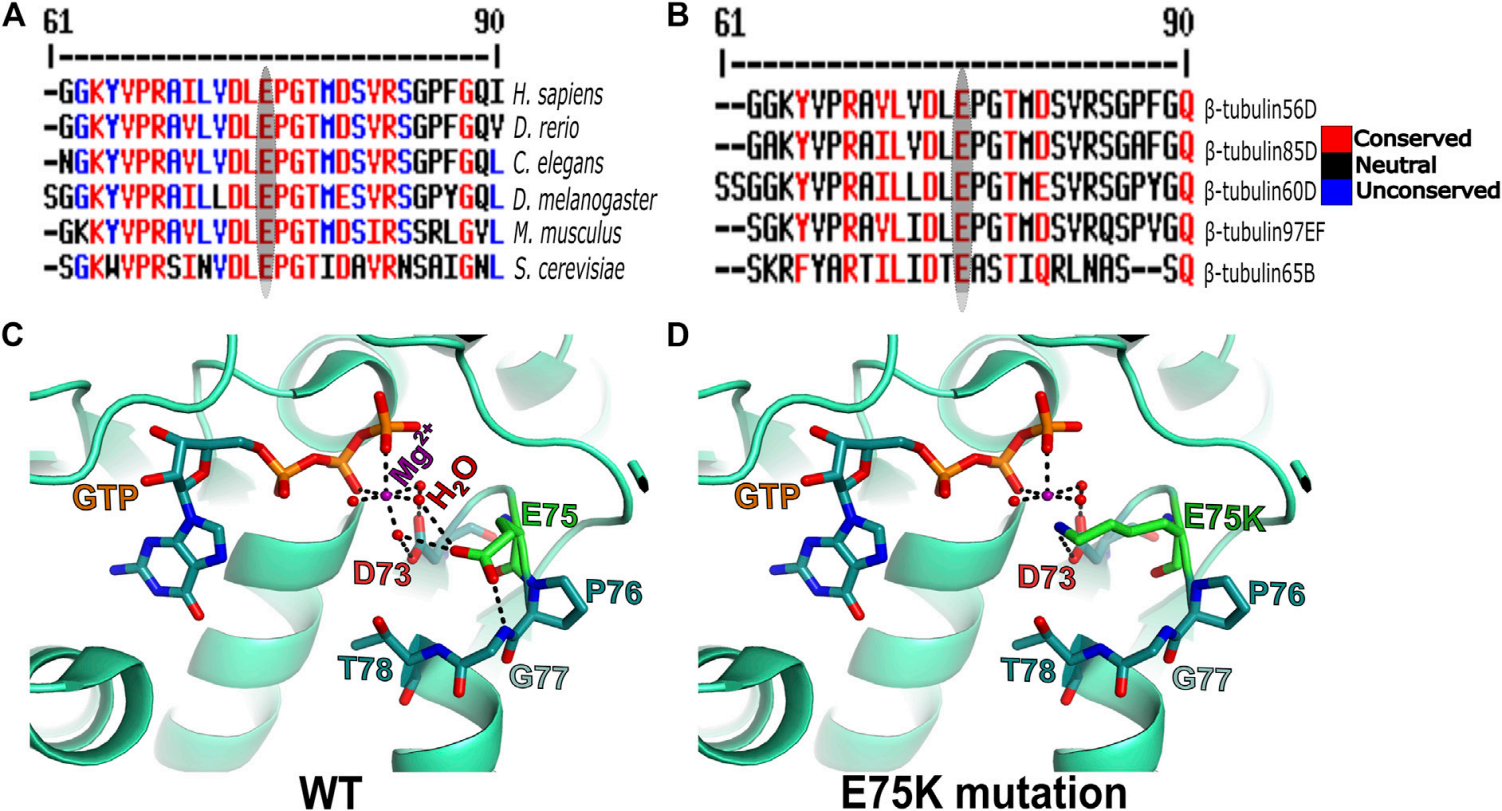
Amino acid conservation and structural modeling. Color scheme showing the sequence alignment of orthologues of *ß-tubulin60D* from different eukaryotes **(A)** and sequence alignment among all the known *Drosophila ß*-tubulin isoforms **(B)**. The conservation scoring is performed by MultAlin. The scoring scheme works from 0 for the least conserved alignment position up to 10 for the most conserved alignment position, as indicated by the color assignments. The amino acid residue, glutamate {E}, is highly conserved among all the organisms ranging from human to *Drosophila*. The *ß*-tubulin isoforms in *Drosophila* also show a similar degree of conservation. The conservation is highlighted with an ellipse. Structural comparison of wild-type *ß*-*tubulin60D* protein **(C)** and the *Pin1* mutant *ß*-*tubulin60D* protein **(D)** shows how the single amino acid change at position 75 {E75K} affects the magnesium binding capacity of the protein, thereby affecting its functions.

To get better insight into the molecular nature of the *Pin* ^*1*^ allele, we sequenced the *β-tubulin60D* coding region from the genomic DNA of *Pin* ^1^/Df flies and compared the sequence to that of the WT strain/allele. The sequencing revealed a missense mutation in Pin1/Df at base pair 223 of its mRNA resulting in a glutamate-to-lysine replacement at position 75 (E75K). Alignment of the *Drosophila ß*-tubulin60D protein across organisms from humans to yeast revealed that E75 is highly evolutionarily conserved (Figure 5A). Moreover, the alignment of *Drosophila β-tubulin60D* protein with the other four *Drosophila β-tubulin* paralogs (*β-tubulin56D, β-tubulin65B, β-tubulin85D,* and *β-tubulin97EF*) also revealed higher conservation of this specific glutamate (Figure 5B). The *Drosophila ß*-tubulin60D protein contains the highly conserved N-terminal guanine nucleotide-binding region, intermediate domain (paclitaxel binding site), and C-terminal domains that constitute the binding surface for MAPs and molecular motors such as kinesins and dynein. By analyzing the *ß*-tubulin structure (*Drosophila melanogaster*, Tubulin beta-1 chain, PDB: 6TIY), the newly identified *β-tubulin60D* mutation (E75K) lies at the guanine nucleotide-binding region. In this position, E75 acts as an alpha helix N-cap stabilizing residue via its hydrogen bond to the alpha-helix backbone (Figure 5C). Additionally, E75 creates hydrogen bonds with two water molecules which are part of the Mg^2+^ hydration shell. This magnesium is critical for the GTP-Mg^2+^ complex binding at the tubulin nucleotide-binding site (Figure 5D).

### 3.4 Microtubule Network is Mis-Organized in *Pin* ^*1*^ Mutant

**FIGURE 6:**
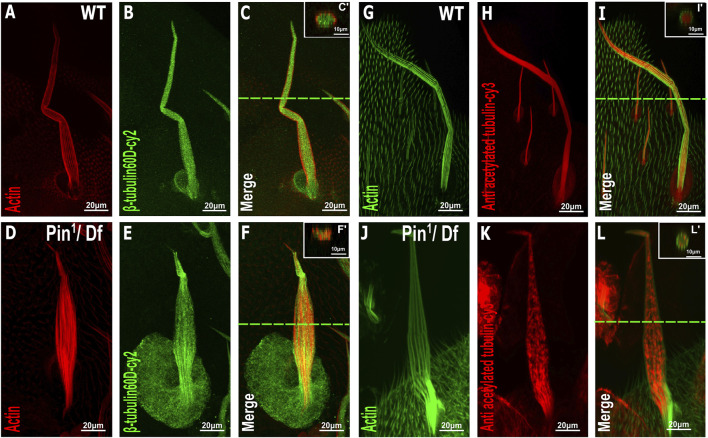
Distribution of β-tubulin and acetylated α-tubulin is affected in the Pin1 mutant bristle. Confocal projections of bristles of ∼37 h APF from WT **(A–C)** and *Pin* ^*1*^/Df **(D–F)** pupae stained with red-phalloidin (red) and with anti-β-tubulin60D antibodies (green). Digital cross-sections marked by a green line of wild-type **(C′)** and *Pin* ^*1*^/Df **(F′)** pupae demonstrate a gradual decrease in *ß*-tubulin60D density at the middle of the bristle shaft. Confocal projections of bristles of ∼38 h APF from WT **(G–I)** and *Pin* ^*1*^/Df **(J–L)** pupae stained with green-phalloidin (green) and with anti-acetylated tubulin-antibodies (red). Digital cross-sections marked by a green line of wild-type **(I′)** and *Pin* ^*1*^/Df **(L′)** pupae demonstrate a patchy and uneven distribution in acetylated *a*-tubulin density throughout the bristle shaft compared to that of the wild-type. APF–after prepupa formation.

To further examine the effects of *Pin* ^*1*^ on bristle shaft development, we characterized the organization of MTs in the bristle using antibodies against *ß*-tubulin60D (Leiss et al., 1988) and against acetylated tubulin (Figures 6G–L), which recognize stable MT network in the bristle (Bitan et al., 2012). Anti-β-tubulin60D staining revealed that the *ß*-tubulin60D protein is present in *Pin* ^*1*^ hemizygous pupae (Figures 6D–F). This observation suggests that the E75K alteration found in *Pin* ^*1*^ does not significantly affect the stability of the *ß*-tubulin60D protein; still, this staining showed that MTs are extremely disorganized (Compare Figures 6A–C to Figures 6D–F). Whereas in WT pupae, MTs are found throughout the bristle shaft, in hemizygous *Pin* ^*1*^ mutants, they are not evenly distributed and often appear as aggregates found at various locations along the bristle shaft. Disorganization of the stable *a*-tubulin MT network was also evident in the hemizygous *Pin* ^*1*^ mutants] (Compare Figures 6G–I to Figures 6J–L).

## 4 Discussion

### 4.1 *β-tubulin60D* is not an Essential Gene

This is the first study where a well molecularly defined protein null allele of *βTub60D* was generated and characterized. This well-characterized *βTub60D* allele demonstrated unambiguously that *βTub60D* is not an essential gene. These results disagree with previous studies in which multiple alleles of βTub60 were generated, which showed lethality at different stages of development, from embryogenesis to larval stages (Kimble et al., 1990; Dettman et al., 1996; Dettman et al., 2001). Using an ethyl methanesulfonate (EMS) or diepoxybutane (DEB) mutagen screen led to the identification of one larval lethal complementation group of five alleles of *β-tubulin60D*, called-*β*3t1–*β*3t5, and some, but not all, of these alleles, could be rescued by a *β-tubulin60D* transgene. Examination of the homozygous and transhetrozygous phenotype suggested that *β-tubulin60D* is required for viability and fertility (Kimble et al., 1990). In the second screen, eight new alleles of *β-tubulin60D* were identified; six were induced by EMS, one by gamma radiation, and one by P-element mutagenesis. All alleles were recessive lethal in the larval stages, except for two semi-lethal but sterile alleles (Dettman et al., 1996). Some of the combinations of the transheterozygous alleles also exhibited bristle and flight defects. All of the *β-tubulin60D* alleles that were generated by EMS were not protein null (Dettman et al., 1996). Sequencing one of the *β-tubulin60D* allele, β3t2, which belongs to the class I severe alleles, revealed no lesion in the coding region of the gene (Dettman et al., 2001). To this end, the β3t2 allele of *β-tubulin60D* is the only available allele, but we found that it is no longer recessive lethal, and it complements *Pin* ^*1*^ allele bristle defects (data not shown), suggesting that this stock is no longer a βTub60 allele. To conclude, the facts that all other lethal alleles are not well molecularly characterized and also not available for further characterization, together with the fact that our molecularly defined protein null allele of *β-tubulin60D* revealed that *β-tubulin60D* is not an essential gene, led us to conclude that *β-tubulin60D* is not required for *Drosophila* viability.

### 4.2 *Pin* ^*1*^ Encodes a Novel Dominant-Negative Allele of the *β-tubulin60D* Gene

To further characterize the *βTub60D* gene, we found that *Pin* ^*1*^, an uncharacterized mutation with a dominant bristle defects, is a novel dominant negative allele of the *β-tubulin60D* gene. First, our protein-null *βTub60D* alleles fail to complement the bristle defect found in hemizygous *Pin* ^*1*^ mutants. Second, the expression of *βTub60D* protein specifically in the bristle completely rescues the bristle defect found in hemizygous *Pin* ^*1*^ mutants. Third, as expected from the gene part of the MT lattice, the MT network in bristles from transhetrozygous and hemizygous *Pin* ^*1*^ mutants is severely affected. Fourth, our genetic analysis showed a missense mutation in Pin1/Df at base pair 223 of its mRNA, resulting in an amino acid replacement from glutamate at position 75 to lysine (E75K). Bioinformatic analysis suggests that replacing the glutamate with lysine residue destabilizes the alpha helix since lysine is an alpha helix N-cap destabilizing residue. In addition, lysine’s positive charge will be located near the Mg^2+,^ which might prevent its binding or, in general, alter the GTP-Mg^2+^ complex binding capabilities.

In humans, mutations in *β-tubulin* genes are associated with defects in neuronal development (Jaglin et al., 2009; Poirier et al., 2010; Tischfield et al., 2010; Breuss et al., 2012; Poirier et al., 2013; Simons et al., 2013; Cushion et al., 2014), oocyte meiosis (Feng et al., 2016; Sha et al., 2020), thrombocytopenia (Fiore et al., 2017), and macrothrombocytopenia (Davis et al., 2008; Kunishima et al., 2009). All these human mutations are found as heterozygous missense mutations, suggesting that either haploinsufficiency or a dominant-negative mechanism cause these diseases. Study on the disease-associated mutations in *TUBB3* showed that R62Q, A302T, R380C, or R262C mutations impair tubulin heterodimer formation *in vitro*. The R62Q, R262H, R262C, A302T, and E410K mutations also disrupted microtubule dynamics in yeast. The E410, D417, and R262 mutations affect Kinesin binding to MT (Tischfield et al., 2010). These results suggest that these missense mutations do not affect *TUBB3* protein stability, but affect the cellular function of the MT network, maybe due to the “toxic” effect of the mutant tubulin isotype. However, the debate on the potential mechanisms for the disease-causing heterozygous tubulin mutants is still open. Our study showed that a complete loss of function of *βTub60D* does not affect fly viability, with no other obvious defects. The fact that the *βTub60D* null allele had no defects in *Drosophila* development, although it has tissue-specific expression, suggests that other *βTub* paralogues may compensate for the loss of the *βTub60D* gene. On the other hand, we demonstrated that *Pin* ^*1*^ is a heterozygous missense allele of the *βTub60D* gene with a tissue-specific requirement. Thus, the fact that a complete loss of *βTub60D* had no apparent defects in *Drosophila* development and that *Pin* ^*1*^ is a heterozygous missense allele of the *βTub60D* supports the idea that a dominant-negative rather than a haploinsufficient mechanism underlies the function of the *Pin* ^*1*^ allele.

## Data Availability

The mass spectrometry proteomics data have been deposited to the ProteomeXchange Consortium via the PRIDE [1] partner repository with the dataset identifier PXD030317.
