# Supplementary material for: Revisiting the Role of ß-Tubulin in Drosophila Development: β-tubulin60D is not an Essential Gene, and its Novel Pin 1 Allele has a Tissue-Specific Dominant-Negative Impact
Source: Front Cell Dev Biol. 2022 Jan 17;9:787976. doi: 10.3389/fcell.2021.787976 (PMC8802551; doi:10.3389/fcell.2021.787976)
Supplement: Supplementary file 1 [file Table1.docx]

| **Genotype** | **Average no. of progeny from 24 hr egg collection** | **% relative fertility** |
| --- | --- | --- |
| WT ♂ x WT ☿ | 98.33±0.7 | 100 ^a^ |
| CRISPR KO *β-Tubulin60D^M^* ♂ x WT ☿ | 94.66±1.1 | 96.2 ^a^ |
| CRISPR KO  *β-Tubulin60D^M^* ☿ x WT ♂ | 87.66±1.3 | 89.1 ^a^ |

**Supplementary Table 1**. ***β-tubulin60D* null allele does not affect male and female fertility**. Tukey's test for post hoc analysis shows that the percentage of relative fertility has no significant difference when compared statistically to the control group. ^a^ Same letter in the column indicates no significant statistical difference between the groups.
