# Supplementary figures and images for: Revisiting the Role of ß-Tubulin in Drosophila Development: β-tubulin60D is not an Essential Gene, and its Novel Pin 1 Allele has a Tissue-Specific Dominant-Negative Impact
Source: Front Cell Dev Biol. 2022 Jan 17;9:787976. doi: 10.3389/fcell.2021.787976 (PMC8802551; doi:10.3389/fcell.2021.787976)

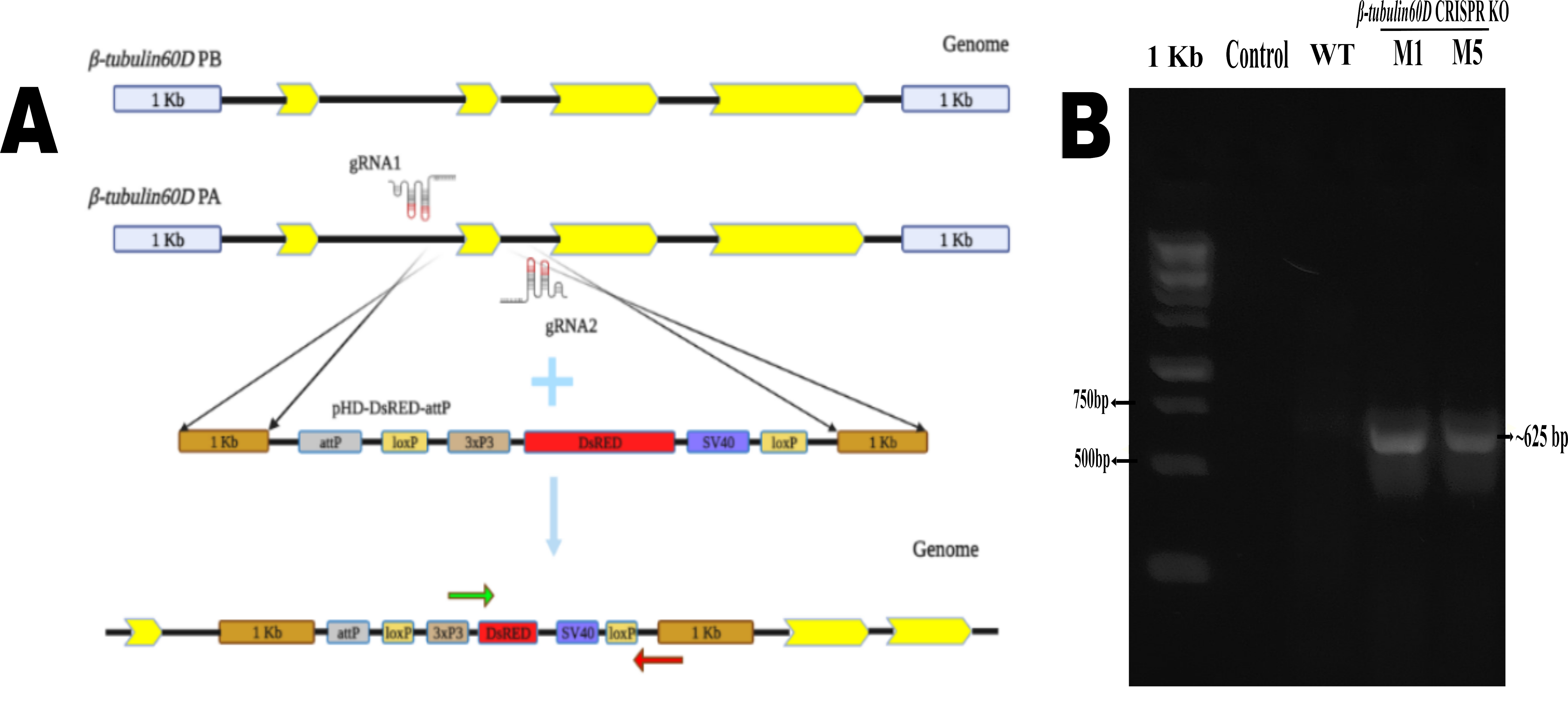

Supplement: Supplementary file 2 [file Image1.JPEG]
